# Adverse childhood experiences and depressive symptoms among lesbian and bisexual women in China

**DOI:** 10.1186/s12905-023-02686-5

**Published:** 2023-12-19

**Authors:** Biao Zhu, Chenchang Xiao, Changmian Ding, Hong Yan, Liyin Wang, Qingqing Jiang, Jiawei Tian, Liqing Wei

**Affiliations:** 1 School of Public Health, Wuhan University, Wuhan, China; 2 Anhui Provincial Center for Disease Control and Prevention, Hefei, China; 3 Wuhan City College, Wuhan, China; 4 Department of Medical Record, The People’s Hospital of Dehong, Dehong, China

**Keywords:** Adverse childhood experiences, Depressive symptoms, Lesbian and bisexual women

## Abstract

**Background:**

Despite the relationship between Adverse childhood experiences (ACEs) and depressive symptoms, which has been well researched in general populations, little is known about homosexual and bisexual populations, especially lesbian and bisexual women in China. This study aims to investigate the prevalence of ACEs and depressive symptoms and to analyze the relationship between them among lesbian and bisexual women in China.

**Methods:**

The eligible participants were aged 16 years or older who report their sexual orientation as homosexual or bisexual. The data was collected through anonymous questionnaires with the help of Lespark in Beijing from July 18 to December 29, 2018, and all participants had informed consent to this study. Univariate analysis and multiple linear regression analyses were performed to explore the relationship between ACEs and depressive symptoms among lesbian and bisexual women. All statistical analyses were conducted by the software of SPSS 22.0.

**Results:**

Among 301 lesbian and bisexual women, 81.4% were lesbian, 18.4% were bisexual women, and the majority were 21–30 years. As for ACEs, 51.5% reported at least one ACE, in which emotional neglect (22.6%) and emotional abuse (22.3%) were common ACEs. As for depressive symptoms of lesbian and bisexual women, the detection rate was 56.1%. The multiple linear regression analyses showed that abuse (*β* = 2.95, 95%*CI:*1.07–4.83) and neglect (*β* = 3.21, 95%*CI*:1.09–5.31) were positively associated with depressive symptoms and lesbian and bisexual women with three (*β* = 4.11, 95%*CI*: 0.99–7.22) or more (*β* = 6.02, 95%*CI*: 3.23–8.78) ACEs suffered from more depressive symptoms than others.

**Conclusion:**

Adverse childhood experiences (ACEs) and depressive symptoms were at high prevalence among lesbian and bisexual women in China. ACEs were associated with depressive symptoms, especially childhood abuse and neglect experiences that have a significant effect on lesbian and bisexual women mental health.

## Background

Adverse Childhood Experiences(ACEs) are identified as traumatic events, including all types of abuse, neglect and household dysfunction that early adverse life experiences [1]. Studies have confirmed that early life experiences have an impact on health throughout the life course [2, 3]. ACEs can bring about the increasing risk of poor health outcomes, e.g. substance abuse [2], poor physical health-related indices [1], socially unacceptable behaviors [1, 4], anxiety [5], and symptoms of depression [6, 7]. Studies abroad have shown a correlation between ACEs and mental health, especially among general population [8, 9] and the sexual minorities [10]. Additionally, some studies had shown that, compared with bisexual women, lesbians and female bisexual had suffered more ACEs [11, 12]. However, few studies on the relationship between ACEs and depressive symptoms directly focused on lesbian and bisexual women, especially in China.

As a type of marginalized sexual minority group, lesbian and bisexual women are less and tend to hide [13, 14], resulting in difficulties to find them. For instance, a survey on adult sexual orientation conducted in the United States showed that lesbians and bisexual women accounted for 1.3% and 2.4% of all surveyed women, respectively [14]. And, among the women aged 15 to 24, 2.7% were lesbians and 7.4% were bisexual [15] in a cross-sectional survey conducted in Shanghai, China. Additionally, it has been reported that about 1/3 of HIV new cases are homosexual men [16], and due to the higher HIV infection rate [17, 18], men who have sex with men (MSM) are focused by more and more research, while less attention has been paid to lesbian and bisexual women. And besides the same stress from traditional marriage and family outlook as MSM, lesbians are more likely to have a crisis on low self-identity because of their unique sensitivity and social pressure [19]. At the same time, lesbians also suffer from anxiety, depression, high-risk sexual behavior, and substance abuse, which greatly threaten their physical and mental health and the quality of life [20, 21]. Besides, as a traditional developing country, people here have poor acceptance of homosexuals, which leads to their concealment of lesbian identity and marginalization of society [22, 23] in China. And lesbian organizations are still very weak and lack of relevant resources in China [19, 24], which makes it difficult to provide effective, convenient, and fast protection for lesbian and bisexual women.

Several studies have shown that, compared with heterosexual women, lesbians have a greater likelihood (OR = 2.4, 95%*CI* = 2.0–2.9) of suffering from depressive symptoms [20]. People with long-lasting depressive symptoms are more likely to have suicidal ideation [25] and high-risk sexual behaviors [26], which give rise to the heavy social burden and the spread of sexually transmitted diseases. Additionally, depressive symptoms are also associated with physical and mental health, such as peptic ulcer, high blood pressure, type 2 diabetes, hyperthyroidism, hypothyroidism, and decline of immune function [27]. Therefore, it is vital to explore the prevalence of ACEs and its impact on depressive symptoms among lesbian and bisexual women in China [12].

## Methods

### Participants and procedure

The cross-sectional study was conducted from July 18 to December 29, 2018, with the help of lesbian organization Lespark in Beijing, China. Women aged 16 or older and self-reported homosexual or bisexual identities were eligible in this study. The internet questionnaires were sent to participants recruited through routine testing services, outreach activities, or peer recommendation after they provided the informed consent. The online help may obtain from trained investigators if participants had any questions during filling out the questionnaires. The questionnaires would be checked once being submitted. After completing the questionnaires, every respondent would get 50 RMB(approximately US$8) for her participation. This study was approved by the Medical Ethics Committee at Wuhan University.

Three hundred seven of the 335 participants completed and submitted the questionnaires (response rate: 91.64%), while 6 were deleted for their heterosexual identities, yielding 301 were regarded as the final sample for statistical analysis.

## Measures

### Demographic characteristics

Age, ethnic (Han and others), education, registration (urban and rural), employment, residence status, monthly income, and sexual orientation were collected in this study. Age was categorized as ≤ 20, 21–25, 26–30, and > 30 years. Education included high school or lower, college or undergraduate, and master or higher. Employment was divided into the student, full-time jobs, part-time jobs, or others. The categories of residence status included living with family, living with female friends, living in a dormitory, and living alone. The monthly income included four categories (e. g. ≤ 3000, 3001–6000, 6001–9000, and > 9000 (RMB). Sexual orientation was asked with the four options (homosexual/bisexual/heterosexual/undecided), and those respondents answering homosexual and bisexual were included in the analysis.

### Depressive symptoms

Depressive symptoms were measured by the Centers for Epidemiological Studies Depression Scale (CESD-10) [28]. The scale including 10 items is often applicable to assess frequency of depressive symptoms in the past 7 days. It is a four-point rating scale, ranging from 0 (never) to 3(always). The total score ranges 0–30, and higher score indicates higher level of depressive symptoms. Individual scoring 10 or higher will be considered to have depressive symptoms. The Cronbach's Alpha of CESD-10 was 0.75 in this study.

#### ACEs

The questionnaire developed in the Kaiser-CDC study [28, 29] was aimed to assess the ACEs of participants. The 10-item scale includes three dimensions, abuse (emotional, physical, and sexual), neglect (emotional and physical), and household challenges (mother treated violently, household substance abuse, mental illness in the household, parental separation or divorce, and criminal household member). There are two options for each item: “Yes’’ and “No”. Participants would be considered to experience the adverse event if they respond “Yes”, and participants were considered to have ACE if they self-reported at least one ACE. Similarly, they were considered to have abuse, neglect, or household challenges if they self-reported at least one of them. ACE scores were acquired by accumulating the number of ACE exposure, which ranged from 0–10. In analysis, ACE scores were categorized as 0, 1, 2, 3, ≥ 4. The Cronbach's Alpha of ACE was 0.74 in this study.

### Statistical analysis

Descriptive analysis was applied to describe demographic characteristics and prevalence of ACEs. Univariate analysis including t-test, one-way ANOVA were performed to explore the bivariate correlations among characteristics, ACEs, and depressive symptoms. Multiple liner regression analysis was used to examine the relationship between ACEs and depressive symptoms. In model 1, the relation between any ACE and depressive symptoms was examined. In model 2, the relation between ACE categories (abuse, neglect, and household challenges) and depressive symptoms was analyzed. In model 3, the relation between ACE scores and depressive symptoms was analyzed. The demographic characteristics with *P* value less than 0.05 in the univariate analysis were included in all multiple liner regression analysis as covariates. All statistical analysis were conducted by the software of SPSS 22.0.

## Results

Among 301 lesbian and bisexual women, 70.7% were 21–30 years, and the majority (94.0%) were Han. 74.1% had a college or undergraduate education, and 83.1% came from urban areas. 66.8% had a full-time job and 23.9% were students. Most of the participants lived with family (30.9%), and 22.3% reported living with female friends, 23.6% living in the collective dormitory, and 23.3% living alone, respectively. The proportion of monthly income in 3001–6000 was the highest (39.9%) followed by ≤ 3000 (25.9%). 81.4% were identified as homosexual while 18.6% were identified as bisexual (See Table 1).

**Table 1:** Characteristics of respondents

Variable	N	%
Age (years)		
≤ 20	44	14.6
21–25	116	38.5
26–30	97	32.2
> 30	44	14.6
Ethnic		
Han	283	94.0
other	18	6.0
Education		
High school or lower	50	16.6
College or undergraduate	223	74.1
Master or higher	28	9.3
Registration		
Urban	250	83.1
Rural	51	16.9
Employment		
Student	72	23.9
Full-time jobs	201	66.8
Part-time jobs/others	28	9.3
Residence		
Family	93	30.9
Female friend	67	22.3
Collective dormitory	71	23.6
Alone	70	23.3
Monthly income		
≤ 3000	78	25.9
3001–6000	120	39.9
6001–9000	64	21.3
> 9000	39	13.0
Sexual orientation		
Homosexuality	245	81.4
Bisexuality	56	18.6

### ACEs among lesbian and bisexual women

Table 2 displays the ACEs status of lesbian and bisexual women. 51.5% reported at least one ACE, of which 24.6% reported one ACE, 9.6% reported two, 7.3% reported three, and 10.0% reported four or more. Among 10 ACEs, emotional abuse (22.3%) and emotional neglect (22.6%) were common adverse experiences in childhood, followed by parental separation or divorce (16.3%).

**Table 2:** ACEsof lesbian and bisexual women

Variable	N	%
Any ACE		
Yes (ACE ≥ 1)	155	51.5
ACE categories		
Abuse		
Emotional abuse	67	22.3
Physical abuse	37	12.3
Sexual abuse	32	10.6
Neglect		
Emotional neglect	68	22.6
Physical neglect	9	3.0
Household challenge		
Parental separation or divorce	49	16.3
Mother treated violently	11	3.7
Household substance abuse	19	6.3
Household mental illness	30	10.0
Incarcerated household member	21	7.0
ACE scores		
0	146	48.5
1	74	24.6
2	29	9.6
3	22	7.3
≥ 4	30	10.0

### Depressive symptoms of lesbian and bisexual women

The average score of CESD-10 was 11.60 (SD = 7.35), ranging from 0 to 30. The detection rate of depressive symptoms was 56.1%. Table 3 found that depressive symptoms were different among lesbian and bisexual women of different age (*F* = 3.076, *P* = 0.028), education (*F* = 3.759, *P* = 0.024), employment(*F* = 5.518, *P* = 0.004), and residence (*F* = 5.178, *P* = 0.002).

**Table 3:** Differences in depressive symptoms in lesbian and bisexual women with different characteristics

Variable	M ± SD	t/F	*P*
Age (years)		3.076	0.028
≤ 20	10.80 ± 7.15		
21–25	12.12 ± 7.76		
26–30	10.26 ± 6.41		
> 30	14.00 ± 7.85		
Ethnic		-0.866	0.387
Han	11.51 ± 7.38		
Other	13.06 ± 6.88		
Education		3.759	0.024
High school or lower	12.44 ± 7.75		
College or undergraduate	11.86 ± 7.35		
Master or higher	8.07 ± 5.72		
Registration		-0.216	0.829
Urban	11.56 ± 7.49		
Rural	11.80 ± 6.68		
Employment		5.518	0.004
Student	11.10 ± 7.22		
Full-time job	11.18 ± 7.16		
Part-time job/others	15.93 ± 7.87		
Residence		5.178	0.002
Family	12.31 ± 7.82		
Female friend	8.85 ± 5.93		
Collective dormitory	11.41 ± 7.37		
Alone	13.49 ± 7.27		
Monthly income		1.068	0.363
≤ 3000	12.08 ± 7.50		
3001–6000	12.10 ± 7.27		
6001–9000	11.11 ± 7.40		
> 9000	9.92 ± 7.19		
Sexual orientation		0.981	0.327
Homosexuality	11.80 ± 7.43		
Bisexuality	10.73 ± 6.96		

Results in Table 4 reveals that lesbians exposing to ACE were inclined to experience a higher level of depressive symptoms (*t* = -3.684, *P* < 0.001). Especially those individuals who experienced abuse (*t* = -4.732, *P* < 0.001) and neglect (*t* = -5.090, *P* < 0.001) had a higher level of depressive symptoms. Lesbian and bisexual women with higher ACE scores were more likely to have a higher level of depressive symptoms (*F* = 7.935, *P* < 0.001).

**Table 4:** Differences in depressive symptoms in lesbian and bisexual women with different ACEs

Variable	M ± SD	t/F	*P*
Any ACE		-3.684	< 0.001
Yes	13.08 ± 7.79		
No/	10.03 ± 6.52		
ACE category			
Abuse		-4.732	< 0.001
Yes	14.71 ± 7.84		
No/	10.25 ± 6.71		
Neglect		-5.090	< 0.001
Yes	15.65 ± 7.74		
No/	10.40 ± 6.79		
Household challenge		-1.518	0.131
Yes	12.65 ± 8.27		
No/	11.15 ± 6.88		
ACE score		7.935	< 0.001
0	10.03 ± 6.52		
1	11.39 ± 7.54		
2	11.83 ± 6.67		
3	14.41 ± 8.68		
≥ 4	17.47 ± 7.16		

Table 5 displays the results from multiple linear regression analyses controlling for demographic characteristics. The results in model 1 indicated that for each increase in reported ACE, there was an increase in reported symptoms of depression (*β* = 2.42 (95% CI:0.82–4.01) independent of age, education, employment status and residence.. Results in model 2 demonstrated that those minorities having any abuse or neglect experience were more likely to have depressive symptoms. Likewise, results from model 3 showed that compared with individuals who had no ACE, those with 3 ACEs and ≥ 4 ACEs have a higher level of depressive symptoms. In addition, results from three models indicated that compared with individuals who had part-time jobs/others, those with full-time jobs might undergo moderate depressive symptoms. Compared with individuals who lived alone, those lived with their female friends might have a lower level of depressive symptoms. Compared with those with a master's degree or above, those with a bachelor's degree usually have higher depressive symptoms.

**Table 5:** Multiple linear regression of lesbian and bisexual women

Variable	Model 1	Model 2	Model 3
	**β(95%CI)**	**β(95%CI)**	**β(95%CI)**
Age			
≤ 20	-3.72(-7.23,-0.21)	-3.34(-6.73,0.06)	-2.95(-6.43,0.53)
21–25	-1.68(-4.22,0.86)	-1.42(-3.88,1.04)	-1.23(-3.73,1.28)
26–30	-2.42(-4.97,0.13)	-2.18(-4.67,0.30)	-1.98(-4.50,0.54)
> 30(ref.)			
Education			
High school or lower	4.00(0.52,7.49)*	3.31(-0.8,6.69)	3.31(-0.14,6.76)
College or undergraduate	4.01(1.20,6.82)*	3.44(0.72,6.17)*	3.64(0.86,6.41)*
Master or higher(ref.)			
Employment			
Student	-2.91(-6.49,0.66)	-3.17(-6.64,0.30)	-2.46(-5.99,1.07)
Full-time job	-3.62(-6.45,-0.80)*	-3.57(-6.33,-0.82)*	-3.20(-6.00,-0.40)*
Part-time (ref.)			
Residence			
Family	-0.98(-3.17,1.21)	-0.56(-2.69,1.57)	-0.80(-2.95,1.36)
Female friend	-4.33(-6.70,-1.97)**	-4.16(-6.46,-1.87)**	-4.25(-6.58,-1.92)**
Collective dormitory	-1.30(-3.98,1.38)	-0.83(-3.45,1.79)	-1.52(-4.16,1.12)
Alone (ref.)			
ACE			
Any ACE			
Yes	2.42(0.82,4.01)*		
No(ref.)			
ACE category			
Abuse			
Yes		2.95(1.07,4.83)*	
No(ref.)			
Neglect			
Yes		3.21(1.09,5.31)*	
No(ref.)			
Household dysfunction			
Yes		-0.72(-2.49,1.05)	
No(ref.)			
ACE score			
0(ref.)			
1			1.05(-0.87,2.97)
2			1.38(-1.36,4.11)
3			4.11(0.99,7.22)*
≥ 4			6.02(3.24,8.79)**

^a^Model 1, the relation between any ACE and depressive symptoms was examined. Model 2, the relation between ACE categories (abuse, neglect, and household challenges) and depressive symptoms was analyzed. Model 3, the relation between ACE scores and depressive symptoms was analyzed

^b^*P < 0.05, **P < 0.01

## Discussion

This study found that 51.5% of the respondents had at least one ACE, which was equal to that of MSM reported by Ding (51.4%) [30] and higher than that of the general population reported by Edwards (34.6%) [31], Lin (34.16%) [32] and Eleonora lob (24%) [33]. Among all types of ACEs, the reported rates of emotional neglect and emotional abuse among participants were higher, accounting for 22.6% and 22.3%, respectively. It was not in accordance with the findings among heterosexual women that most (20.4%) reported experiencing physical abuse ever [34]. It was also found that 10.6% of participants reported ever suffering from sexual abuse before age 18, which is a bit higher than general Chinese women (8.9%) [35]. However, some studies conducted in the United States showed that the reporting rate of childhood sexual abuse among lesbians was 55.8% and twice as much as that of heterosexual females (26.5%) [36]. No matter among lesbians or heterosexual women, the reported prevalence of childhood sexual abuse in the United States is higher than that of Chinese women. The low proportion of sexual abuse among children in China is possibly due to Chinese traditional cultural backgrounds like Confucian culture and collectivist views [37]. Meantime, Stoltenborgh suggested that cultural values in Asia could prevent childhood sexual abuse victims from disclosing their experiences, especially when the abusers were the victims’ family members because exposing the childhood sexual abuse experience would bring shame on the family [38].

In this study, more than half of lesbian and bisexual women (51.6%) went through depressive symptoms. Similarly, Yi found that 47.2% of Korean lesbians and 59.2% of bisexuals experienced depressive symptoms [39]. However, among general Chinese females, 33.2% reported depressive symptoms [40], which is less than that of lesbian and bisexual women in this study. Kerr [20] and Case [41] also showed in their research that compared with heterosexual women, lesbians have a greater likelihood of depressive symptoms. As a marginal group, lesbians usually face more pressure [42] and are inclined to encounter social discrimination [43] because of their sexual orientation. Exiting studies have confirmed that sexual minority stress and experience of discrimination are gravely related to depressive symptoms and other mental health [42–44].

Multiple linear regression analysis showed that having ACE was positively associated with a higher level of depressive symptoms, which was consistent with previous studies. Chapman suggested that ACEs increase the risk of adults’ depression (this may occur in decades after ACEs) [45]. Also, Cheong reported that there was a close relationship between ACEs and depressive symptoms [46]. In three dimensions of ACE, abuse and neglect experience showed a significant correlation with a higher level of depressive symptoms. It has been reported that the adults who suffered from childhood abuse were more easily develop cognitive bias and negative self-concept and depressive symptoms as well. Lee’s study also showed that childhood emotional abuse can increase the level of depression symptoms in adulthood, and in turn lead to suicidal attempts [47]. Furthermore, this study also found that those with more ACEs have a much higher level of depressive symptoms [48]. Robert Wm Blum’s research [49] showed a similar finding that the more ACE exposures increase, the worse depressive symptoms would be. These findings suggest those lesbian and bisexual women who had ACEs especially abuse and neglect experiences need to be paid more attention to in intervention for improving their mental health. Therefore, it is important to screen for ACEs, recognize their potential effects, and provide specific psychological support for lesbian and bisexual women with ACEs.

Some studies have reported that there is no significant correlation between household dysfunction and mental problems such as depressive symptoms [50, 51], which probably because household dysfunction, neglect, and abuse usually coexist, and neglect and abuse have stronger effects on mental health than household dysfunction. In contrast to that, another study thought that children with family dysfunction experience may form personality characteristics such as inferiority and timidity, and so on, which render children perceive the world negatively, and then easily create depression [8, 52]. In this study, the relationship between household challenges and depressive symptoms in lesbian and bisexual women wasn’t found. It was possible due to the small sample, also possibly because household challenges aren’t really connected with depressive symptoms. Further study with a large sample or prospective studies is needed to ascertain the relationship in the future.

### Limitations

This study had several limitations. Firstly, lesbian and bisexual women in the study were recruited by convenient sampling with the help of a lesbian organization, and the sample size was insufficient, which possibly limited the results. Secondly, the causality could not be directly determined due to the cross-sectional study design. Thirdly, ACEs and depressive symptoms of participants were collected by self-reporting recall bias and reporting bias might be introduced into this study. Lastly, considering the feasibility of electronic questionnaire filling, we mainly recruited lesbian and bisexual women aged 16 and above.

## Conclusions

This study suggests that lesbian and bisexual women have a high prevalence of both ACEs and depressive symptoms, and there is a significant relationship between ACEs and depressive symptoms. It’s necessary to pay more attention to lesbians and bisexuals with ACEs to better improving their mental health.

## Data Availability

The datasets generated and analyzed during the current study are not publicly available due to the confidentiality signed with respondents but are available from the corresponding author on reasonable request.

## Abbreviation

ACEs: Adverse childhood experiences

## References

[1] Bellis MA (2014). Adverse childhood experiences and associations with health-harming behaviours in young adults: surveys in eight eastern European countries. Bull World Health Organ.

[2] Mersky JP, Topitzes J, Reynolds AJ (2013). Impacts of adverse childhood experiences on health, mental health, and substance use in early adulthood: a cohort study of an urban, minority sample in the US. Child Abuse Negl.

[3] Kelly-Irving M (2013). The embodiment of adverse childhood experiences and cancer development: potential biological mechanisms and pathways across the life course. Int J Public Health.

[4] Fox BH (2015). Trauma changes everything: Examining the relationship between adverse childhood experiences and serious, violent and chronic juvenile offenders. Child Abuse Negl.

[5] Sareen J (2013). Adverse childhood experiences in relation to mood and anxiety disorders in a population-based sample of active military personnel. Psychol Med.

[6] Brockie TN (2015). The relationship of adverse childhood experiences to PTSD, depression, poly-drug use and suicide attempt in reservation-based native American adolescents and young adults. Am J Community Psychol.

[7] Hughes K (2017). The effect of multiple adverse childhood experiences on health: a systematic review and meta-analysis. Lancet Public Health.

[8] Blosnich JR, Andersen JP (2015). Thursday's child: the role of adverse childhood experiences in explaining mental health disparities among lesbian, gay, and bisexual US adults. Soc Psychiatry Psychiatr Epidemiol.

[9] Poole JC, Dobson KS, Pusch D (2017). Anxiety among adults with a history of childhood adversity: Psychological resilience moderates the indirect effect of emotion dysregulation. J Affect Disord.

[10] Ding CM (2019). Association of adverse childhood experience and attention deficit hyperactivity disorder with depressive symptoms among men who have sex with men in China: moderated mediation effect of resilience. Bmc Public Health.

[11] Balsam KF, Rothblum ED, Beauchaine TP (2005). Victimization over the life span: a comparison of lesbian, gay, bisexual, and heterosexual siblings. J Consult Clin Psychol.

[12] Alvy LM (2013). Sexual identity group differences in child abuse and neglect. J Interpers Violence.

[13] Praeger R (2019). The prevalence and factors associated with smoking among lesbian and bisexual women: analysis of the Australian national drug strategy household survey. Int J Drug Policy.

[14] Caceres BA (2019). Cardiovascular disease disparities in sexual minority adults: an examination of the behavioral risk factor surveillance system (2014–2016). Am J Health Promot.

[15] Lian QG (2015). Sexual orientation and smoking history: results from a community-based sample of youth in Shanghai, China. Environ Health Prevent Med.

[16] Halkitis PN, Figueroa RP (2013). Sociodemographic characteristics explain differences in unprotected sexual behavior among young HIV-negative gay, bisexual, and other YMSM in New York City. AIDS Patient Care STDS.

[17] Yan HC (2020). The central role of nondisclosed men who have sex with men in human immunodeficiency virus-1 transmission networks in Guangzhou, China. Open Forum Infect Dis.

[18] van Kesteren NMC, Hospers HJ, Kok G (2007). Sexual risk behavior among HIV-positive men who have sex with men: a literature review. Patient Educ Couns.

[19] Zhang C (2019). Analysis of the role cognition and related factors of lesbians in formal marriage. Chin J Hum Sex.

[20] Kerr DL, Santurri L, Peters P (2013). A comparison of lesbian, bisexual, and heterosexual college undergraduate women on selected mental health issues. J Am Coll Health.

[21] Fredriksen-Goldsen KI (2013). Health disparities among lesbian, gay, and bisexual older adults: results from a population-based study. Am J Public Health.

[22] Hua BY, Yang VF, Goldsen KF (2019). LGBT older adults at a crossroads in mainland china: the intersections of stigma, cultural values, and structural changes within a shifting context. Int J Aging Hum Dev.

[23] Ren ZJ, Yuan C (2018). Mental health professionals' ethical dilemma when working with gay men who are in heterosexual marriages in China. J Gay Lesbian Ment Health.

[24] Mai Z (2011). The status quo of lesbianism and the difficulties faced by the lesbian movement——Taking Xi'an province as an example. Popul Dev.

[25] Zatti C (2017). Childhood trauma and suicide attempt: a meta-analysis of longitudinal studies from the last decade. Psychiatry Res.

[26] Liu Y (2014). Research on the Correlation of Discrimination, Mental Health Status and Unsafe Sexual Behaviors among HIV/AIDS Patients. J Cent South Univ (Medical Science).

[27] Yang NX (2018). Influence of oncology nurses' empathy on lung cancer patients' cellular immunity. Psychol Res Behav Manag.

[28] Felitti VJ (1998). Relationship of childhood abuse and household dysfunction to many of the leading causes of death in adults. The Adverse Childhood Experiences (ACE) Study. Am J Prev Med.

[29] Prevention, C.f.D.C.a. Adverse childhood experiences (ACE) study. 2013 [cited 12 Apr 2019]; Available from: http://www.cdc.gov/ace/about.htm.

[30] Ding C (2019). Association of adverse childhood experience and attention deficit hyperactivity disorder with depressive symptoms among men who have sex with men in China: moderated mediation effect of resilience. BMC Public Health.

[31] Edwards VJ (2003). Relationship between multiple forms of childhood maltreatment and adult mental health in community respondents: results from the adverse childhood experiences study. Am J Psychiatry.

[32] Jia Z (2020). Cumulative exposure to adverse childhood experience: depressive symptoms, suicide intensions and suicide plans among senior high school students in Nanchang City of China. Int J Environ Res Public Health.

[33] Iob E, Steptoe A (2019). Adverse childhood experiences, inflammation, and depressive symptoms in later life: a prospective cohort study. Lancet.

[34] Stoddard JP, Dibble SL, Fineman N (2009). Sexual and physical abuse: a comparison between lesbians and their heterosexual sisters. J Homosex.

[35] Ma YD (2018). Prevalence of Childhood Sexual Abuse in China: A Meta-Analysis. J Child Sex Abus.

[36] Wilsnack SC (2012). Characteristics of childhood sexual abuse in lesbians and heterosexual women. Child Abuse Negl.

[37] Ji K, Finkelhor D, Dunne M (2013). Child sexual abuse in China: a meta-analysis of 27 studies. Child Abuse Negl.

[38] Stoltenborgh M (2011). A global perspective on child sexual abuse: meta-analysis of prevalence around the world. Child Maltreat.

[39] Yi H (2017). Health disparities between lesbian, gay, and bisexual adults and the general population in South Korea: rainbow connection project I. Epidemiol Health.

[40] Qian JH, Li NX, Ren XH (2017). Obesity and depressive symptoms among Chinese people aged 45 and over. Sci Rep.

[41] Case P (2004). Sexual orientation, health risk factors, and physical functioning in the Nurses' Health Study II. J Womens Health.

[42] Meyer IH (2003). Prejudice, social stress, and mental health in lesbian, gay, and bisexual populations: conceptual issues and research evidence. Psychol Bull.

[43] Katz-Wise SL, Hyde JS (2012). Victimization experiences of lesbian, gay, and bisexual individuals: a meta-analysis. J Sex Res.

[44] Katz-Wise SL (2017). Associations of timing of sexual orientation developmental milestones and other sexual minority stressors with internalizing mental health symptoms among sexual minority young adults. Arch Sex Behav.

[45] Chapman DP (2004). Adverse childhood experiences and the risk of depressive disorders in adulthood. J Affect Disord.

[46] Cheong EV (2017). Adverse childhood experiences (ACEs) and later-life depression: perceived social support as a potential protective factor. BMJ Open.

[47] Lee MA (2015). Emotional abuse in childhood and suicidality: the mediating roles of re-victimization and depressive symptoms in adulthood. Child Abuse Negl.

[48] Wu L (2013). Childhood abuse experience and the relationship between cognitive bias and depression. Chin J Clin Psychol.

[49] Blum RW, Li M, Naranjo-Rivera G (2019). Measuring adverse child experiences among young adolescents globally: relationships with depressive symptoms and violence perpetration. J Adolesc Health.

[50] Negriff S (2020). ACEs are not equal: Examining the relative impact of household dysfunction versus childhood maltreatment on mental health in adolescence. Soc Sci Med.

[51] Finkelhor D (2015). A revised inventory of adverse childhood experiences. Child Abuse Negl.

[52] You Y (2017). The influence of family function on suicidal ideation of middle school students: a moderated mediation effect. Chin J Clin Psychol.

